# Effects of *Laminaria japonica* polysaccharides on exercise endurance and oxidative stress in forced swimming mouse model

**DOI:** 10.1186/s40709-016-0049-4

**Published:** 2016-04-26

**Authors:** Feiwei Yan, Haitao Hao

**Affiliations:** Public PE Department, Zhejiang Shuren University, No. 8 Shuren Street, Hangzhou, 310015 Zhejiang Province People’s Republic of China; Department of Physical Education and Military Training, China Jiliang University, Hangzhou, 310018 China

**Keywords:** *Laminaria japonica* polysaccharides, Forced swimming test, Exercise endurance, Oxidative stress, Mice

## Abstract

**Background:**

Polysaccharides are the major active ingredients responsible for the bioactivities of *Laminaria japonica*. However, the effects of *L. japonica* polysaccharides (LJP) on exercise endurance and oxidative stress have never been investigated. Therefore, this study was conducted to investigate the effects of LJP on exercise endurance and oxidative stress in a forced swimming mouse model. The animals were divided into four groups, namely the control (C), LJP-75, LJP-150, and LJP-300 groups, which received physiological saline and 75, 150, and 300 mg kg^−1^ LJP, respectively, by gavage once a day for 28 days. This was followed by a forced swimming test and measurements of various biochemical parameters.

**Results:**

LJP increased swimming time to exhaustion, the liver and muscle glycogen content, and levels of superoxide dismutase, glutathione peroxidase, and catalase in the serum, liver, and muscle, which were accompanied by corresponding decreases in the malondialdehyde (MDA) content of the same tissues. Furthermore, decreases in blood lactic acid and serum myeloperoxidase (MPO) levels were observed.

**Conclusion:**

LJP enhanced exercise endurance and protected mice against exhaustive exercise-induced oxidative stress.

## Background

The generation of reactive oxygen species (ROS) is a necessary and unavoidable consequence of aerobic metabolism [1]. The enhanced oxygen consumption during exercise leads to an increased flux of oxygen through the mitochondria, and 2–5 % of this oxygen is not completely reduced to water and, therefore, generates ROS [2]. Under normal physiological conditions, cells have adequate defenses against ROS production and enough endogenous enzymatic and nonenzymatic antioxidant reserves [3, 4]. However, during strenuous physical exercise, the rate of ROS generation exceeds that of their removal and oxidative stress occurs [5]. Consequently, accumulated excessive ROS can attack vital biomolecules such as plasma membrane lipids and proteins and, thereby, deteriorate normal cellular functions and further contribute to muscle damage [6]. Specifically, it has been shown that strenuous physical exercise decreases antioxidants levels and increases lipid peroxidation markers in the blood and tissues [7]. Therefore, antioxidant supplementation may protect against exhaustive exercise-induced oxidative stress by forming less active radicals or quenching free radicals and ROS [8]. Many antioxidant bioactive compounds, such as polysaccharides from *Radix pseudostellariae* [*Pseudostellaria heterophylla* (Miq.) Pax], polysaccharides from *Auricularia auricula*, polysaccharides from *Cordyceps sinensis* mycelium, polysaccharides from *Ganoderma lucidum*, salidroside, ginsenoside-Rg_1_, ginsenoside‑Rb_1_, flavonoid from *Citrus limon* (L.) Burm. F. as well as polyphenols from *Vaccinium corymbosum* L., have been reported for their protective effects on exhaustive exercise-induced oxidative stress [4, 9–16].

The brown seaweed *Laminaria japonica*, is a common seafood consumed in China and numerous other countries and has been documented as a drug in traditional Chinese medicine (TCM) [17]. In the ancient literature, *L. japonica* has been recorded as an important therapeutic agent for phlegm elimination, detumescence, and weight loss for more than 1000 years [18]. Over the past decades, *L. japonica* has been the focus of attention of chemists and pharmacologists because of its abundant functional compound content and the associated biological properties. The major active constituents of *L. japonica* are polysaccharides including alginate, fucoidan, and laminarin [19]. Recent studies have demonstrated that *L. japonica* polysaccharides (LJP) have a wide range of biological properties including anti-apoptosis, antivirus, anticoagulant, antitumor, antithrombotic, anti-radiation, hypoglycemic, hypolipidemic, and immunostimulatory [20–23]. Furthermore, LJP protected endogenous antioxidant enzymes, inhibited lipid peroxidation, and exhibited high antioxidant activities including the oxygen radical absorbance capacity (ORAC), 2,2ʹ-azino-bis-(3-ethylbenzthiazoline-6-sulfonic acid) (ABTS) and reduced power tests [24, 25], suggesting that LJP might reduce exhaustive exercise-induced oxidative stress. Therefore, the current study aimed to demonstrate the protective effects of LJP against exercise endurance and oxidative stress in a forced swimming mouse model.

## Results and discussion

### Effects of LJP on swimming time to exhaustion of mice

**Fig. 1:**
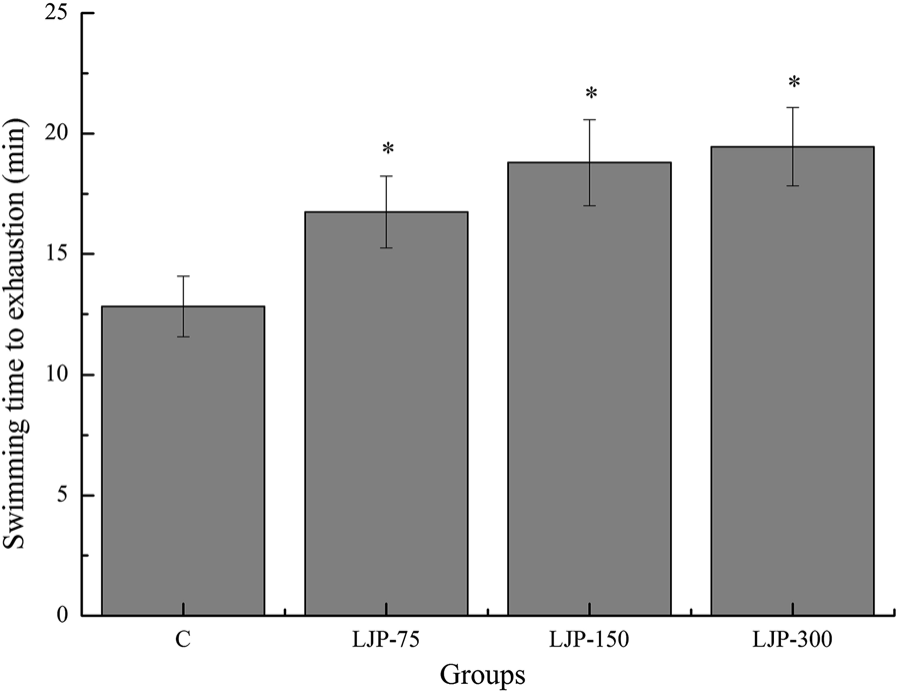
Effects of *L. japonica* polysaccharides on swimming time to exhaustion of mice. Data are mean ± standard deviation (SD); n = 12, *p < 0.05 compared with control (C) group

Exercise endurance is an important parameter for evaluating anti-fatigue treatments, and the forced swimming test has been widely used for this purpose with high reproducibility [26]. The lengths of the swimming time to exhaustion indicate the degree of exercise tolerance and fatigue. As shown in Fig. 1, swimming time to exhaustion of the LJP-75, LJP-150, and LJP-300 groups were significantly longer than that of the control (C) group (*p* < 0.05) with increased rates of 30.58, 45.57, and 51.72 %, respectively. This result indicates that LJP enhanced the exercise endurance and had anti-fatigue effects.

### Effects of LJP on blood lactic acid levels of mice

**Fig. 2:**
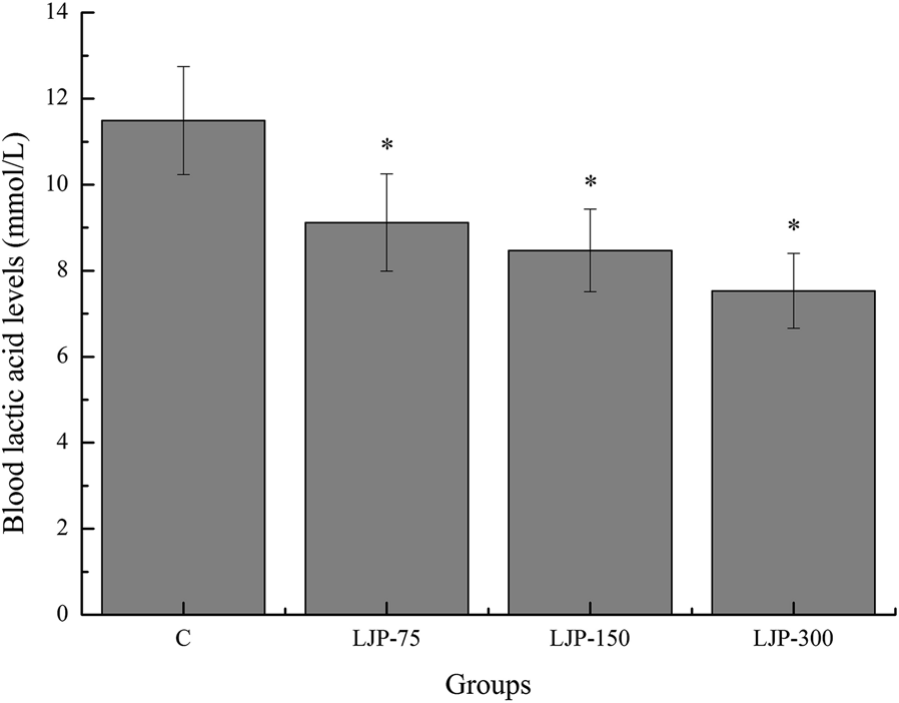
Effects of *L. japonica* polysaccharides on blood lactic acid levels of mice. Data are mean ± standard deviation (SD); n = 12, *p < 0.05 compared with control (C) group

The blood lactic acid (BLA) level tends to increase during strenuous physical exercise because anaerobic metabolism becomes the dominant energy-producing mechanism. The increased lactic acid level further reduces the pH, which could induce various biochemical and physiological effects including glycolysis, phosphofructokinase, and calcium ion release through muscular contraction [27]. Therefore, BLA is an important marker for evaluating the degree of fatigue of a living organism. As shown in Fig. 2, BLA levels of the LJP-75, LJP-150, and LJP-300 groups were significantly lower than that of the C group (*p* < 0.05), and the decrease rates were 25.99, 35.65, and 52.58 %, respectively. This result indicates that LJP effectively inhibited and lowered BLA production and, thereby, retarded the occurrence of fatigue.

### Effects of LJP on glycogen contents of the liver and muscle of mice

**Fig. 3:**
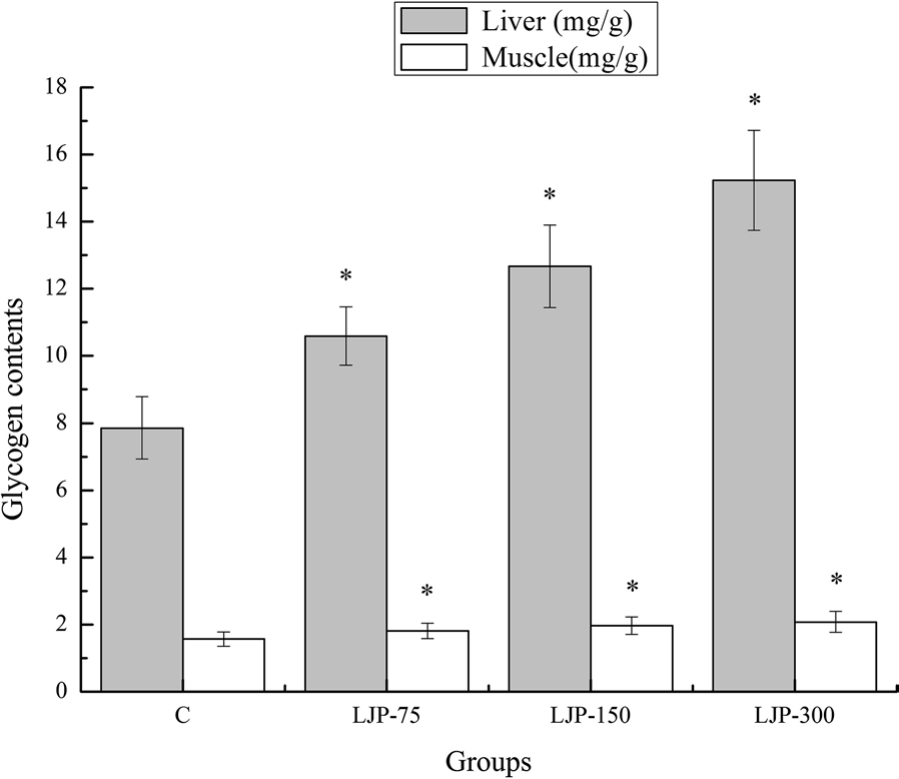
Effects of *L. japonica* polysaccharides on glycogen contents of liver and muscle of mice. Data are mean ± standard deviation (SD); n = 12, *p < 0.05 compared with control (C) group

Energy for exercise is derived initially from the breakdown of glycogen in muscle which may be depleted during strenuous exercise, and at later stages, the energy is derived from the liver glycogen [10, 28]. The depletion of liver glycogen leads to the onset of fatigue [12]. As shown in Fig. 3, the glycogen contents of the liver and muscles of the LJP-75, LJP-150, and LJP-300 groups were significantly higher than that of the C group (*p* < 0.05). Specifically, the increased rates in the liver were 34.73, 61.19, and 93.76 %, respectively, while in muscle, 15.29, 25.48, and 32.48 %, respectively. The results indicate that LJP reduced the liver and muscle glycogen consumption by improving its reserve or reducing the consumption during exercise. Furthermore, the anti-fatigue effects of LJP might be related to the improved metabolic control of exercise and activation of energy metabolism [29].

### Effects of LJP on superoxide dismutase (SOD), glutathione peroxidase (GPx), and catalase (CAT) levels of mouse serum, liver and muscle

It was previously demonstrated that antioxidant enzymes play a significant role in protecting the body against ROS [15]. The principal antioxidant enzymes include superoxide dismutase (SOD), glutathione peroxidase (GPx), and catalase (CAT), and they act to reduce ROS [30]. Regular physical exercise has been shown to increase antioxidant enzyme activities in the blood and tissues of humans and animals [31, 32], which can be attributed to a compensatory response to counteract the possible detrimental effects associated with oxidative stress [33]. However, other studies have reported that strenuous exercise causes a dramatic drop in antioxidant enzyme activities in the blood and tissues [14]. Although inconsistent findings have been reported on the level of antioxidant enzymes, it appears that the variation of these enzymes is dependent not only on the type of tissues measured but also on the mode and intensity of exercise [15].

**Fig. 4:**
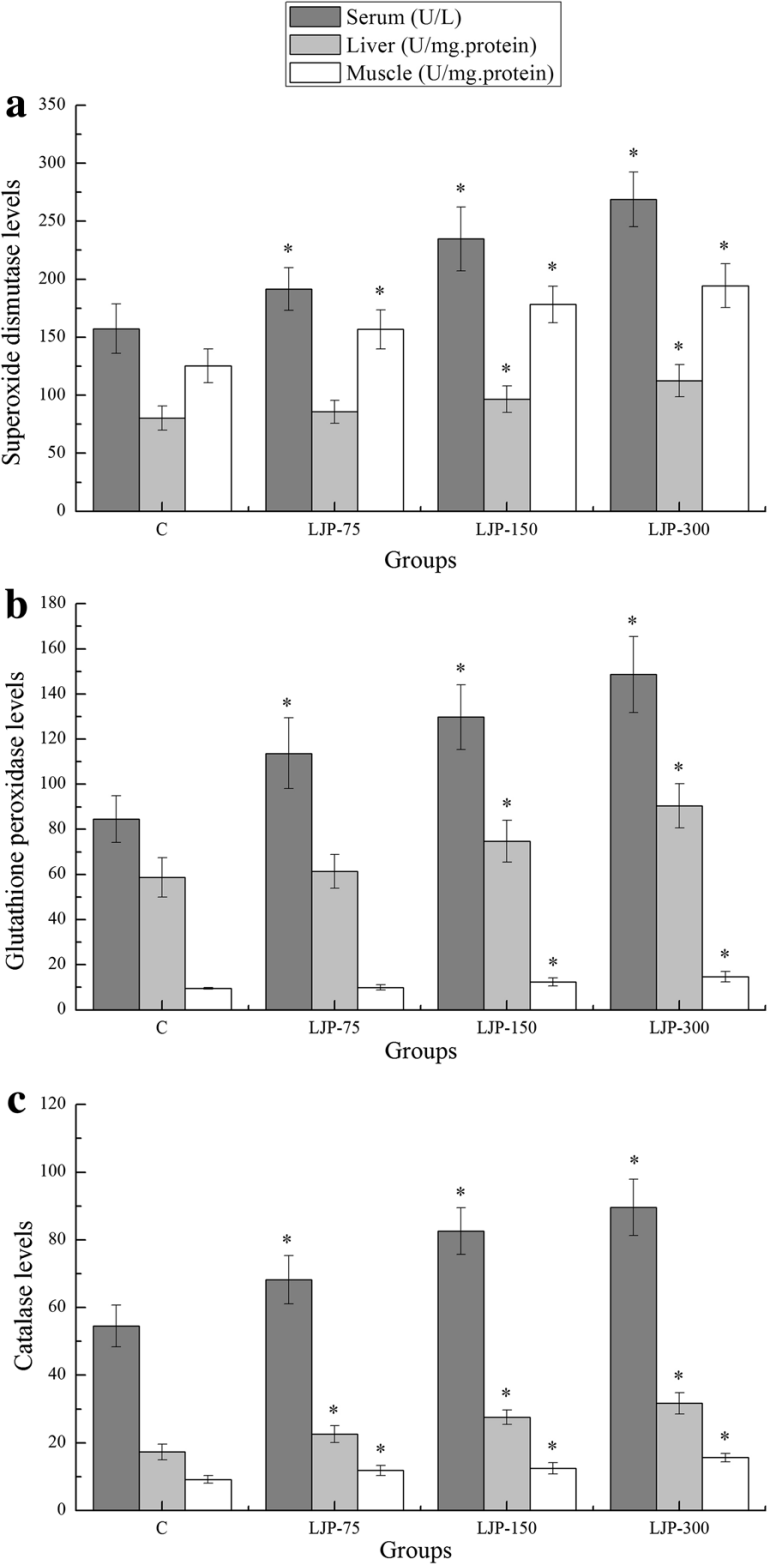
Effects of *L. japonica* polysaccharides on superoxide dismutase (SOD), glutathione peroxidase (GPx), and catalase (CAT) levels of mouse serum, liver and muscle. Data are mean ± standard deviation (SD); n = 12, *p < 0.05 compared with control (C) group

Previous studies have demonstrated that the variation in the levels of antioxidant enzymes in different tissues might be due to tissue-specific metabolic differences [34]. The liver is a critical physiological metabolic organ in organisms, involved in almost all of the substance metabolism, and contains higher levels of antioxidant enzymes than other tissues, which in turn release more ROS with increased lipid peroxidation products [35, 36]. Recent studies have demonstrated a tissue-specific expression of GPx and CAT, with their highest activities occurring in the liver [4, 12, 34]. As shown in Fig. 4a, SOD serum and muscle levels of the LJP-75, LJP-150, and LJP-300 groups were significantly higher than that of the C group (*p* < 0.05) with serum increased rates of 21.73, 49.10, and 70.81 %, respectively, and muscle increased rates of 24.88, 42.05, and 54.87 %, respectively. Furthermore, the SOD levels in the liver of the LJP-150 and LJP-300 groups were significantly higher than that of the C group (*p* < 0.05), with increased rates of 20.09 and 40.02 %, respectively. Although the liver SOD level of the LJP-75 group also increased, no significant difference was observed (*p* > 0.05). As shown in Fig. 4b, the serum GPx levels of the LJP-75, LJP-150, and LJP-300 groups were significantly higher than that of the C group (*p* < 0.05), with increased rates of 34.44, 53.44, and 75.82 %, respectively. In addition, the liver and muscle GPx levels of the LJP-150 and LJP-300 groups were significantly higher than that of the C group (*p* < 0.05). Moreover, the increase rates for the LPJ-75 and LPJ-150 groups were 27.29 and 53.91 %, respectively in the liver, while the muscle rates were 30.87 and 55.81 %, respectively. Although the liver and muscle GPx levels of the LJP-75 group also increased, no significant difference was observed (*p* > 0.05). As shown in Fig. 4c, the serum, liver, and muscle CAT levels of the LJP-75, LJP-150, and LJP-300 groups were significantly higher than that of the C group (*p* < 0.05). Moreover, the increased ratios in the serum were 25.15, 51.37 and 64.26 %, respectively; in the liver, 30.35, 58.92, and 83.04 %, respectively; and in the muscle 28.89, 35.61, and 70.25 %, respectively. The results indicate that LJP upregulated the main antioxidant enzyme activity, which might protect against exhaustive exercise-induced oxidative stress. However, further research needs to be carried out to elucidate this hypothesis.

### Effects of LJP on malondialdehyde (MDA) content of mouse serum, liver and muscle

**Fig. 5:**
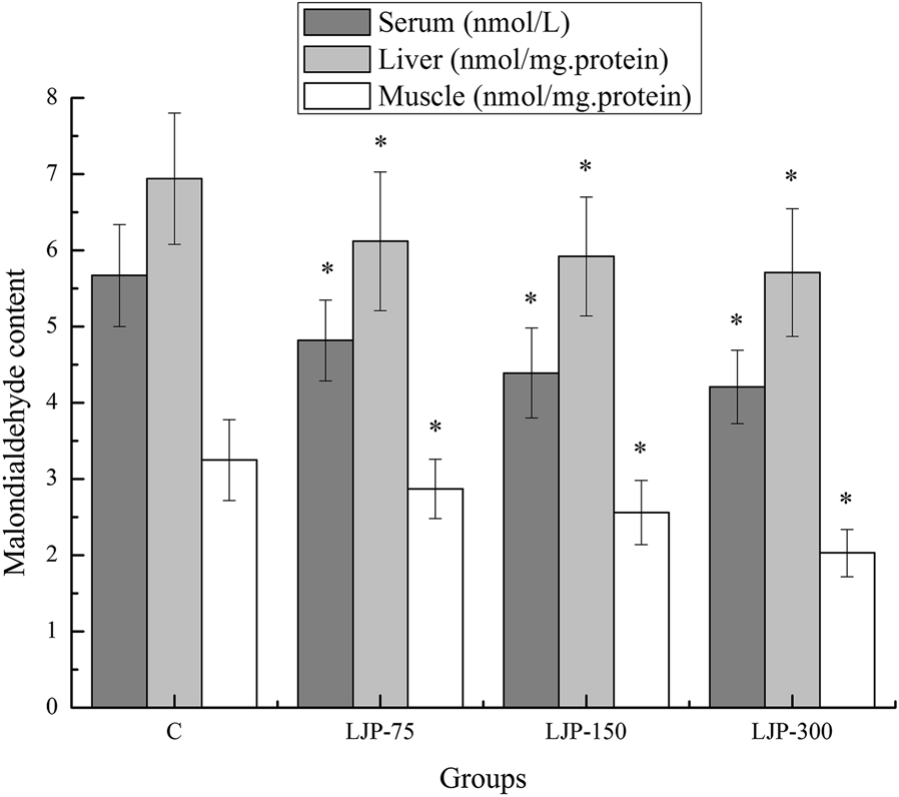
Effects of *L. japonica* polysaccharides on malondialdehyde (MDA) content of mouse serum, liver and muscle. Data are mean ± standard deviation (SD); n = 12, *p < 0.05 compared with control (C) group

Strenuous physical exercise increases the production of ROS, which consequently attack the membrane lipids and results in lipid peroxidation product formation. Significant increases in lipid peroxidation products in the serum, liver, and muscle after exhaustive exercise have been recorded in several studies [37]. Malondialdehyde (MDA), one of the final products of polyunsaturated fatty acid peroxidation, has been widely investigated in exercise studies as a marker of oxidative stress [38]. As shown in Fig. 5, the MDA content of the serum, liver, and muscle of the LJP-75, LJP-150, and LJP-300 groups were significantly lower than that of the C group (*p* < 0.05). Moreover, the decreased rates in the serum were 17.63, 29.16, and 34.68 %, respectively; in the liver were 13.39, 17.23, and 21.54 %, respectively; and in the muscle were 13.24, 26.95, and 60.09 %, respectively. These results indicate that LJP effectively reduced lipid peroxidation.

### Effects of LJP on serum myeloperoxidase (MPO) levels of mice

**Fig. 6:**
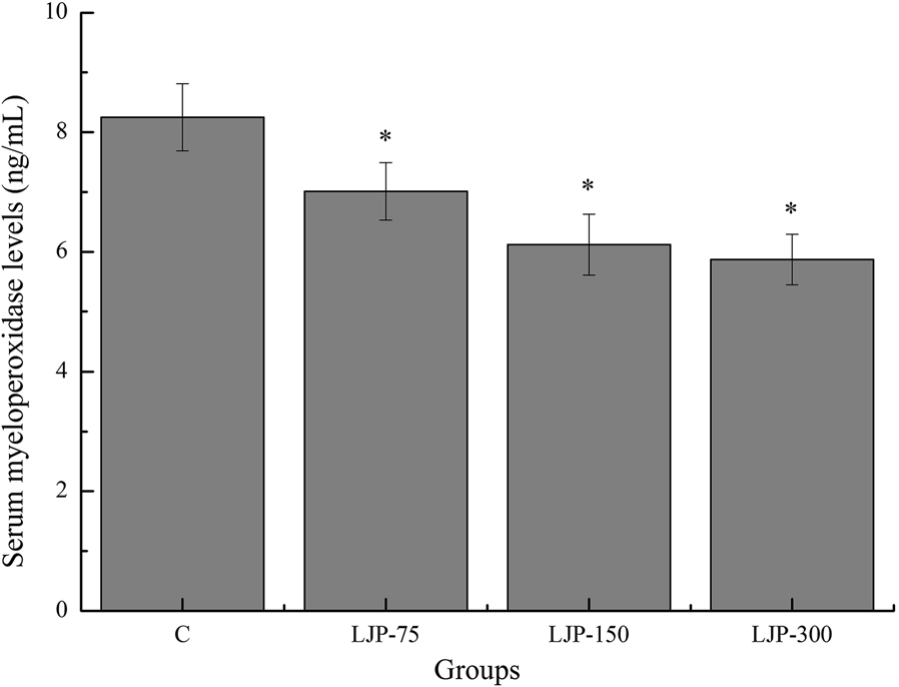
Effects of *L. japonica* polysaccharides on serum myeloperoxidase (MPO) levels of mice. Data are mean ± standard deviation (SD); n = 12, *p < 0.05 compared with control (C) group

Exercise induces neutrophil priming for oxidative activity as evidenced by the increased neutrophil myeloperoxidase (MPO) activity [39]. Accumulating evidence indicates that neutrophil infiltration into tissues is associated with exhaustive exercise-induced oxidative damage [40, 41]. Neutrophils are capable of further generating free radicals via the action of nicotinamide adenine dinucleotide phosphate oxidase [42]. In addition, neutrophils produce hypochlorite from hydrogen peroxide (H_2_O_2_) by the action of MPO, a marker for neutrophil infiltration into tissues, during the induction of exercise-induced oxidative damage [43, 44]. As shown in Fig. 6, the serum MPO levels of the LJP-75, LJP-150, and LJP-300 groups were significantly lower than that of the C group (*p* < 0.05), and the decreased rates were 17.69, 34.80, and 40.54 %, respectively. These results indicate that LJP played an important role in inhibiting oxidative damage after exhaustive exercise, and the decreased serum MPO levels may be due to an alteration in the intracellular redox status of the neutrophils [41].

## Conclusions

This study demonstrated that LJP enhanced the exercise endurance of mice by increasing swimming time to exhaustion and the glycogen contents of the liver and muscle, as well as decreasing BLA levels. Furthermore, LJP exhibited a protective effect against exhaustive exercise-induced oxidative stress by increasing the serum, liver, and muscle levels of SOD, GPx, and CAT, as well as decreasing serum MPO and the MDA contents of the serum, liver, and muscle. The experimental data have shed some light on the clinical therapeutic potential of LJP against exhaustive exercise-induced oxidative stress. However, further study is required to ascertain the detailed underlying mechanisms of these effects.

## Methods

### Materials

*Laminaria japonica* was collected in Zhoushan, Zhejiang, China in September 2013 and the plant material was identified by Professor M. J. Wang (College of Life Sciences, China Jiliang University, Hangzhou, China). The fresh *L. japonica* samples were immediately washed with water, sun-dried, ground into a fine powder using a mechanical grinder (FZ102, Taisite Instrument Co., Tianjin, China), filtered through a 40-mesh (200-μm) sieve, and then the dried powder was stored at room temperature (20 ± 2 °C) in a desiccator (300 mm, Huaou Industrial Co., Yancheng, China) until further used.

### Chemicals and reagents

Purchased commercial diagnostic kits were used for the determination of BLA (Leadman Biochemistry Technology Co., Ltd., Beijing, China); tissue glycogen, SOD, GPx, and CAT (Jiancheng Biotechnology Institute, Nanjing, China); MDA (Biosino Bio-technology and Science Inc., Beijing, China); and MPO (Jianglai Biochemistry Technology Co., Ltd., Shanghai, China). All other chemicals and reagents were of analytical grade purity, and they were purchased from Hangzhou Chemical Reagent Co., Ltd. (Hangzhou, China), and were used without further purification.

### Preparation of LJP

The LJP was prepared according to previously published method [21, 45] with minor modifications. Briefly, dried powder sample was defatted with anhydrous ethanol at 60 °C for 3 h with stirring and then mixed with distilled water thrice (1:40, w/v) at 90 °C for 2.5 h. The insoluble residue was separated from the aqueous extract by centrifugation (10,640×*g* for 15 min). Then, the combined supernatants were concentrated to a quarter of the original volume by evaporation and deproteinated using the Sevag method [46]. The solution was added to anhydrous ethanol to obtain an ethanol concentration of 80 %, kept overnight, and then filtered. The resulting precipitate was dissolved in water followed by the addition of anhydrous ethanol to a final ethanol concentration of 80 % and then filtered twice. The precipitate was washed sequentially with 95 % ethanol, anhydrous ethanol, and acetone and then lyophilized to obtain the final extract of LJP at a yield of 21.37 % (w/w) of the original *L. japonica* plant material. The dried LJP was dissolved in saline solution just before use.

### Experimental animals

Adult male Kunming mice (*Mus musculus,* Km, with weight 20 ± 2 g) were procured from the Experimental Animal Center of Zhejiang Province. All animals were housed under standard environmental conditions (temperature 21 ± 2 °C; humidity 45 ± 5 %; and 12-h light:dark cycle) with free access to a standard pellet diet and water ad libitum. All animal studies were performed according to the Guide for the Care and Use of Laboratory Animals of the National Institutes of Health (NIH), as well as the guidelines of the Animal Welfare Act. The experimental protocol was approved (approval number: ZJSR2014‑0113) by the Institutional Animal Care and Use Committee (IACUC) at the Zhejiang Shuren University.

### Experimental design

The mice were allowed to acclimatize to the laboratory environment for 1 week prior to the experiments. Then, the animals were assigned randomly to four groups of 12 mice each namely the C, LJP-75, LJP-150, and LJP-300 groups, which were treated with the vehicle (physiological saline) and 75, 150, and 300 mg kg^−1^ of LJP, respectively, for 28 days. The LJP was dissolved in 1.5 mL of the vehicle, and the C group received the same volume of the vehicle as well. The treatments were administered orally by gavage once a day according to the pretest and dose determined during the active screening.

After the final LJP or vehicle treatment, the mice were allowed to rest for 30 min, and then they were subjected to the forced swimming test using the method described by Li et al. [11]. The apparatus used was an acrylic plastic pool (60, 50, and 50 cm in length, width, and height, respectively) filled with fresh water, which was maintained at 25 ± 0.5 °C at a depth of 40 cm. Each mouse was weighted using a lead wire bundle attached to the tail at 5 % of the body weight. Exhaustion was determined by observing the loss of coordinated movements and failure to return to the surface within 10 s.

### Biochemical analyses

Following the forced swimming test, the mice were anaesthetized with absolute ether and the success of the anaesthesia was confirmed by verifying the absence of reflex responses to noxious stimuli. Then, the mice were euthanized by decapitation, blood samples were collected for BLA analysis, and serum was obtained by centrifugation (2000×*g*, 4 °C, 10 min) for the SOD, GPx, CAT, MPO, and MDA analyses. After blood collection, the liver and gastrocnemius muscle tissues were quickly dissected, washed in ice-cold physiological saline, frozen in liquid nitrogen, and stored at −70 °C for the assays of glycogen, SOD, GPx, CAT, and MDA. The measurements were performed according to the recommended procedures provided by the commercial diagnostic kits.

### Statistical analysis

The data obtained were expressed as mean ± standard deviation (SD). The results were analyzed using a one-way analysis of variance (ANOVA) followed by a post hoc Tukey’s test using the statistical package for the social sciences (SPSS) software (version 15.0, SPSS Inc., Chicago, IL, USA). Values were considered significant when *p* < 0.05.

## Abbreviations

LJP: *Laminaria japonica* polysaccharides
BLA: blood lactic acid
SOD: superoxide dismutase
GPx: glutathione peroxidase
CAT: catalase
MDA: malondialdehyde
MPO: myeloperoxidase
